# Novel mechanism of JNK pathway activation by adenoviral E1A

**DOI:** 10.18632/oncotarget.1860

**Published:** 2014-03-25

**Authors:** Vasily S. Romanov, Anna I. Brichkina, Helen Morrison, Tatiana V. Pospelova, Valery A. Pospelov, Peter Herrlich

**Affiliations:** ^1^ Leibniz Institute for Age Research – Fritz Lipmann Institute (FLI), Jena, Germany; ^2^ Institute of Cytology, Russian Academy of Sciences, St. Petersburg, Russia; ^3^ St. Petersburg State University, St. Petersburg, Russia; ^4^ Present address: Institute of Molecular and Cell Biology, A-STAR, Proteos, Singapore

**Keywords:** adenoviral E1A, AP-1 subunit c-Jun, JNK signaling pathway, small GTPase Rac1, ERM family proteins

## Abstract

The adenoviral oncoprotein E1A influences cellular regulation by interacting with a number of cellular proteins. In collaboration with complementary oncogenes, E1A fully transforms primary cells. As part of this action, E1A inhibits transcription of c-Jun:Fos target genes while promoting that of c-Jun:ATF2-dependent genes including *jun*. Both c-Jun and ATF2 are hyperphosphorylated in response to E1A. In the current study, E1A was fused with the ligand binding domain of the estrogen receptor (E1A-ER) to monitor the immediate effect of E1A activation. With this approach we now show that E1A activates c-Jun N-terminal kinase (JNK), the upstream kinases MKK4 and MKK7, as well as the small GTPase Rac1. Activation of the JNK pathway requires the N-terminal domain of E1A, and, importantly, is independent of transcription. In addition, it requires the presence of ERM proteins. Downregulation of signaling components upstream of JNK inhibits E1A-dependent JNK/c-Jun activation. Taking these findings together, we show that E1A activates the JNK/c-Jun signaling pathway upstream of Rac1 in a transcription-independent manner, demonstrating a novel mechanism of E1A action.

## INTRODUCTION

Viruses and other intracellular pathogens exploit cellular processes for their proliferation. The study of such viral “tricks” has helped in numerous cases to reveal cellular mechanisms. In humans, adenoviruses multiply productively, which prevents their inducing cellular transformation, but in rodent cells several strains of adenoviruses cause oncogenic transformation [1]. The adenoviral oncogene, *E1A*, a gene expressed early in the viral life cycle, together with the second adenoviral early gene, *E1B*, fully transforms primary rodent fibroblasts [2-4]. *E1B* can be substituted by an oncogenic version of *Ras*, H-*RasV12* [5, 6]. E1A is however not rodent-specific: combined expression of three oncogenes, adenoviral *E1A*, H-*RasV12* and *Mdm2*, is sufficient to convert normal human diploid fibroblasts into cancer cells [7]. Identifying mechanisms of E1A action is therefore also relevant for the processes leading to human cancer. In addition, it may reveal yet unknown cellular mechanisms of transformation.

The *E1A* gene gives rise to two splice variants. The proteins encoded are historically called 12S E1A and 13S E1A. The 12S splice form of E1A suffices for the transformation process. E1A does not bind to DNA directly but interacts with a large number of cellular proteins that are involved in the regulation of transcription, e.g. with the transcription factors TBP and RUNX3, the coactivators p300, CBP and PCAF, the corepressor CtBP, the cell cycle inhibitors Rb and p21^Waf1^ [8-10].

We and others have previously shown that E1A strongly induces phosphorylation at Ser63/73 of c-Jun [11] and at Thr69/71 of ATF2 [12-14], the subunits of one form of the dimeric transcription factor AP-1. E1A distinguishes between different AP-1 dimers: it downregulates *c-fos* promoter activity [15] and inhibits expression of genes regulated by c-Jun:Fos, while enhancing expression of c-Jun:ATF2 target genes [16, 17]. Increased activity of c-Jun:ATF2 in response to a variety of extracellular stimuli such as UV irradiation or treatment with chemical carcinogens depends on the phosphorylation of the N-terminal serines/threonines of both subunits. Phosphorylation of these subunits is the function of c-Jun N-terminal kinase (JNK) [18].

These data prompted us to ask whether and how E1A could activate the JNK signaling pathway. In the current paper we show that E1A indeed activates JNK, and that a short region of the N-terminus of E1A is required for JNK/c-Jun activation. Interestingly, E1A induces activation of the JNK pathway too fast to be transcription-dependent. The activation must be achieved by interaction of pre-existing protein components. We show that E1A induces activation of Rac1 and of the dependent protein kinase cascade upstream of JNK, MEKK1-MKK4/7. ERM proteins that are required for the activation of small G-proteins [19-25], are also involved in the E1A-dependent activation. We suggest that E1A activates the JNK/c-Jun signaling pathway upstream of Rac1 in a transcription-independent manner.

## RESULTS

### E1A activates JNK without prior protein synthesis

**Figure 1:**
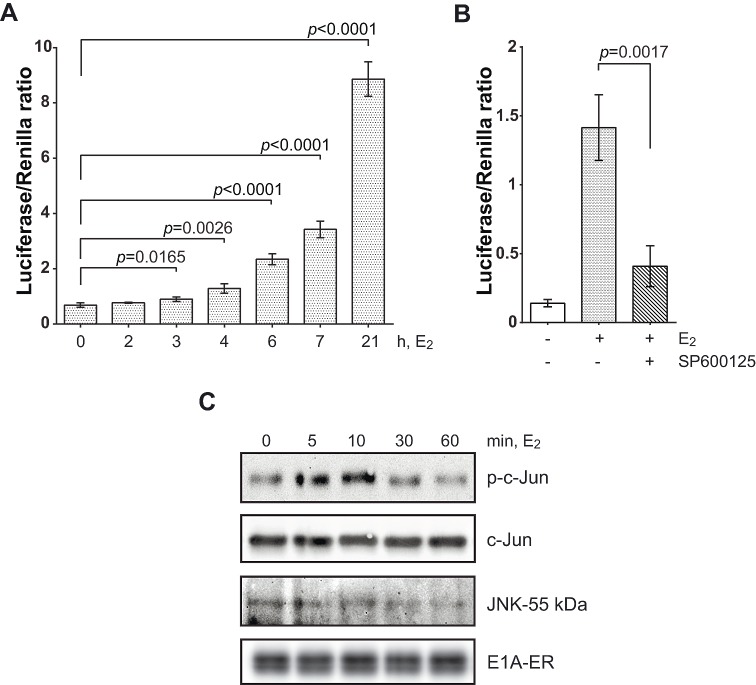
E1A-ER fusion protein activates c-Jun through JNK (A) Histogram showing c-Jun-GAL4 reporter assay. Luciferase activity was determined for the indicated periods of time of incubation with 1 μM E_2_ and then normalized with Renilla. (B) JNK inhibitor SP600125 (10 μM) significantly blocked E1A-induced c-Jun-GAL4 reporter expression. Cells were incubated with E_2_ for 7 h. (C) E1A induced transient JNK activation. *In vitro* protein kinase assay of total JNK was performed with E1A-ER stably transfected cells using KinaseSTAR JNK activity assay kit (see Materials and Methods). In all histograms: one-tailed *p*-values were calculated by Student's t-test; error bars represent s.d.

**Figure 2:**
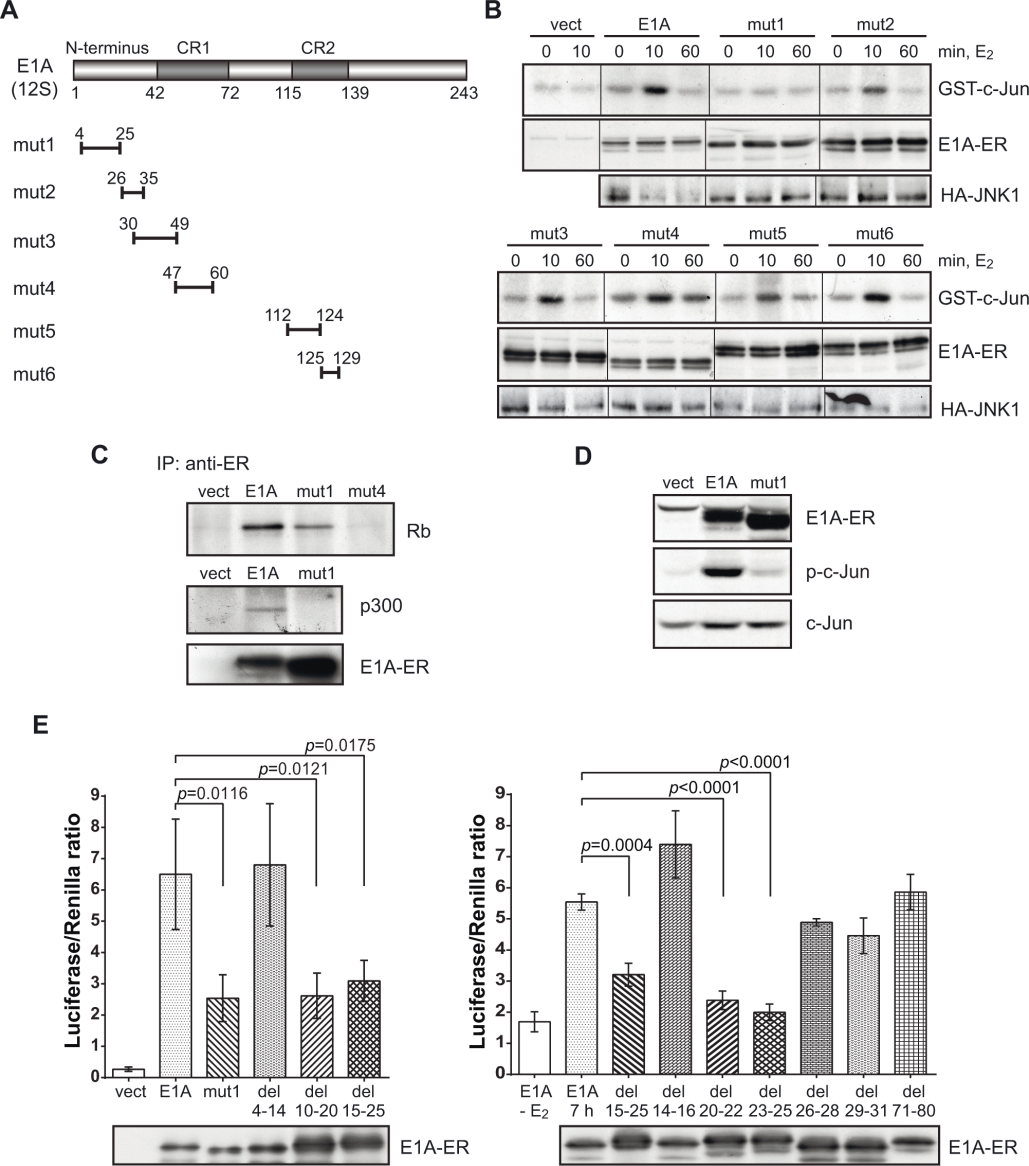
An N-terminal E1A deletion mutant is defective in JNK and c-Jun activation (A) Schematic representation of E1A deletions. The deletion mutants are abbreviated as mut1 to mut6. (B) JNK activation in the deletion mutants determined by radioactive *in vitro* protein kinase assay with GST-c-Jun as a substrate (upper panel) and immunoblots of co-transfected E1A-ER and HA-JNK1 (lower panels). (C) Rb and p300 interaction with wt E1A and mut1. Middle panel: p300 was labeled with ^35^S methionine and visualized by autoradiography. (D) Western blot analysis of phosphorylated (Ser63) c-Jun in E1A-ER stably transfected cells after 10 min of E_2_ treatment. (E) Histogram representing c-Jun-GAL4 reporter assay with the N-terminal deletion mutants of E1A. Cells were incubated with E_2_ for 7 h. One-tailed *p*-values were calculated by Student's t-test; error bars represent s.d. Lower panels: immunoblot of E1A-ER.

**Figure 3:**
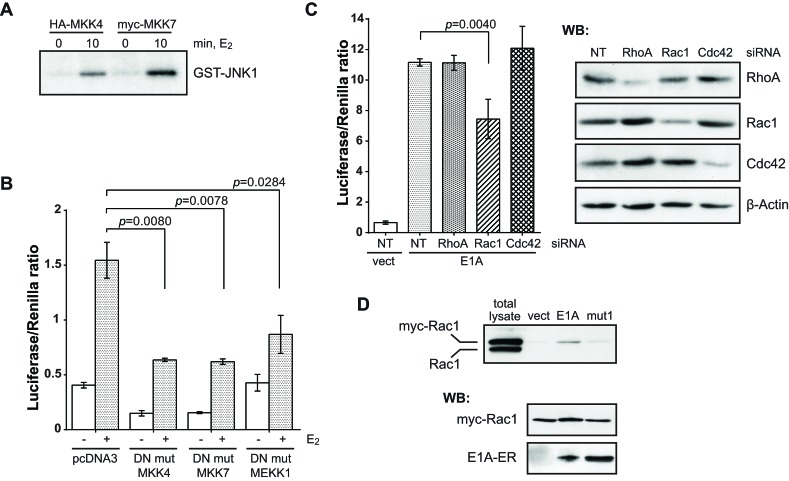
E1A acts upstream of JNK through a Rac1-MEKK1-MKK4/7 pathway (A) E1A activates MKK4 and MKK7. The activities of immunoprecipitated HA-MKK4 and myc-MKK7 were determined by an *in vitro* protein kinase assay with GST-JNK1 as a substrate. (B) c-Jun-GAL4 reporter assay of cells co-transfected with the plasmids encoding for E1A-ER and dominant-negative mutants (DN mut) of JNK upstream signaling components. (C) Histogram showing the c-Jun-GAL4 reporter assay after depletion of RhoA, Rac1 or Cdc42 by siRNA. Right panels: immunoblots of the depleted proteins. (D) Pull-down of GTP-loaded Rac1 with GST-human Pak1-PBD as a substrate. Lower panels: control immunoblots of co-transfected E1A-ER and myc-Rac1. In all histograms: one-tailed *p*-values were calculated by Student's t-test; error bars represent s.d. Cells were incubated with E_2_ for 7 h. NT, NonTarget siRNA.

**Figure 4:**
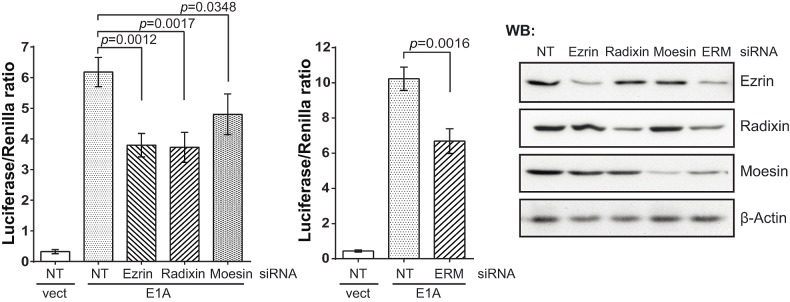
ERM proteins are involved in the E1A-dependent activation of c-Jun siRNA-based depletion of ERM proteins suppresses c-Jun-GAL4 activation determined in the reporter assay. Right panels: immunoblots of the depleted proteins. In the histograms: one-tailed *p*-values were calculated by Student's t-test; error bars represent s.d. Cells were incubated with E_2_ for 7 h. NT, NonTarget siRNA.

In order to investigate the mechanism of c-Jun phosphorylation induced by E1A shown previously [11], we established NIH 3T3 cells with inducible E1A expression. The 12S splice form of E1A was fused with the ligand binding domain of the estrogen receptor (ER) to monitor the immediate effect of E1A activation: the chimeric E1A-ER protein remains inactive in the non-liganded form of the ER and can be activated by estradiol (E_2_) treatment. To measure the activity of c-Jun, we applied a c-Jun-GAL4 reporter assay: c-Jun lacking the basic region/leucine zipper domain and thus unable to dimerize, but retaining the transactivation and phosphorylation domain, was fused to the heterologous DNA-binding domain of the yeast transcription factor GAL4 (GAL_DBD_). Thus, the activity of a reporter gene (luciferase) driven by the GAL4-response element reflected only the activity of c-Jun fused to GAL_DBD_, without interference by other members of the AP-1 transcription factor family. E1A activated by E_2_ indeed stimulated c-Jun-dependent luciferase expression significantly and persistently (Fig. 1A). Control vector-ER plasmid encoding only the ligand binding domain of the ER failed to activate c-Jun (as examples see “vect” in Fig. 2E, 3C, 4), confirming an E1A specific effect, and not an action of the ER domain.

The specific JNK inhibitor SP600125 strongly inhibited E1A-ER-induced reporter expression, suggesting that transactivation of c-Jun by E1A is mediated by JNK activity (Fig. 1B). Moreover, E1A induced JNK activity within 5 min of activation by E_2_, shown by an *in vitro* protein kinase assay using lysates from cells stably transfected with E1A-ER, and using GST-c-Jun as a substrate (Fig. 1C). We conclude that E1A activates JNK fast, obviously without requirement for intermediate protein synthesis. JNK activity was still high at 10 min and then declined.

### An N-terminal E1A deletion mutant is defective in JNK and c-Jun activation

Because E1A interacts with numerous proteins through defined domains, we tested for JNK activation a number of E1A deletion mutants cloned as ER-fusion constructs. Discovery of a defective mutant might help to identify intermediates responsible for the JNK activation. The positions of the first series of deletion mutations of E1A are shown in Fig. 2A. Only one of the mutants, the N-terminal mut1-ER (deletion amino acids 4-25), was unable to induce JNK activity whereas other E1A mutants, mut2 to mut6, induced JNK activity within 10 min of E_2_ treatment (Fig. 2B). This strongly suggests that, an N-terminal binding partner(s) of E1A is responsible for JNK activation.

The N-terminal deletion mutant retained other known E1A functions: mut1-ER could bind one of the known E1A interacting proteins, the retinoblastoma protein (Rb) (Fig. 2C), binding of which is mediated by two E1A domains: amino acid positions 30-60 and 121-127 [10]. Accordingly, Rb interaction was defective in mut4-ER (deletion of amino acid positions 47-60; Fig. 2C). The N-terminal region of E1A is known to be responsible for binding to the coactivator p300 [26]. Expectedly, mut1 cannot bind p300 (Fig. 2C middle panel). Together these data confirmed that E1A-ER proteins were functional and able to form complexes with their known target proteins. Mut3 and mut4 cannot associate with p300, whereas mut3 to mut6 are defective in binding to Rb [26, 27]. These mutants however activate JNK (Fig. 2B) indicating that neither p300 nor Rb are critical elements in this rapid activation of JNK.

Mut1, the only deletion mutant defective in JNK activation, was also defective in the activation of c-Jun that is mediated by JNK-dependent phosphorylation. After 10 min of E_2_ treatment and thus E1A activation, the phosphorylation of c-Jun was detected in stably transfected NIH 3T3 cells by full-length E1A-ER but not by mut1-ER (Fig. 2D). Likewise the transcriptional activity of c-Jun was not enhanced by the E1A mutant defective in JNK activation, shown by measuring c-Jun activity in the Gal_DBD_-reporter assay: while full-length E1A induced reporter activity, mut1 did not (Fig. 2E left panel). Thus, the E1A sequence between amino acid positions 4-25 is critical for JNK and c-Jun activation.

In order to determine the responsible domain more precisely, we generated a number of additional N-terminal deletion mutants, with which we could narrow down the E1A domain required for c-Jun activation (Fig. 2E). Opposite to the deletion mutant 4-14 and despite slightly more efficient transfection, the deletion mutants 10-20 and 15-25 exhibited reduced inducing capability (left panel). Deletion mutants 20-22 and 23-25, but not 14-16 and 26-28, were equally defective in activating c-Jun (right panel). So, we conclude that the domain responsible for c-Jun activation by E1A is amino acids 17-25.

All these results together indicated that a short region of the N-terminus of E1A is required for JNK/c-Jun activation.

### E1A induces activation of the protein kinase cascade upstream of JNK

How does E1A activate JNK? We considered a number of possibilities. First, E1A could directly interact with JNK. Because E1A carries no own protein kinase activity, it might modify JNK such that it became a better substrate for an upstream kinase. Several attempts were made to detect a direct interaction of E1A with JNK by co-immunoprecipitation. However, complexes between E1A and endogenous or co-transfected JNK were not detected (data not shown).

Alternatively, E1A might act on an upstream component that then activated JNK. It is known that JNK is activated predominantly by MKK4 and MKK7 [28]. We measured the activity of both enzymes *in vitro* using GST-JNK1 as a substrate. Within 10 min of E_2_ treatment and thus E1A activation, MKK4 and MKK7 were active and phosphorylated JNK (Fig. 3A). If these kinases were involved in the E1A-dependent activation of the JNK signaling pathway, their dominant-negative mutants should inhibit E1A-dependent JNK activation. Indeed, transfection of dominant-negative MKK4 or MKK7 significantly reduced the E1A-dependent GAL4-c-Jun reporter expression (Fig. 3B). Thus, E1A activates c-Jun through the protein kinase cascade MKK4/7-JNK. Also these kinases could not be co-precipitated with E1A, which prompted us to search further upstream in the cascade for putative E1A targets.

MKK4 and MKK7 in turn are targets of upstream kinases, e.g. MEKK1 [28]. As shown in Fig. 3B, a dominant-negative mutant of MEKK1 interfered with the E1A-induced activation of c-Jun in the Gal_DBD_-reporter assay. Since MEKKs are effectors of the small GTPases RhoA, Rac1 and Cdc42 [28, 29], we examined an involvement of these small G-proteins. We downregulated the synthesis of RhoA, Rac1 or Cdc42 by siRNAs, and confirmed the reduction of their protein levels by Western blot (Fig. 3C, right panels). The downregulation of Rac1, in contrast to that of RhoA and Cdc42, led to reduction of E1A-induced c-Jun activity (Fig. 3C, left panels) approximately to the degree of depletion. These data argue for involvement of Rac1 in the activation of c-Jun by E1A.

### E1A activates Rac1 and requires ERM proteins for c-Jun activation

To directly prove that E1A activates Rac1, we determined the activity level of transfected myc-tagged Rac1 by pull-down of GTP-loaded Rac1, and indeed found E1A-dependent GTP loading (Fig. 3D; note that the activation of Rac1 could only be detected in cells co-expressing E1A-ER and myc-Rac1. Activation of endogenous Rac1 was below detection level probably because of not very high E1A-ER and myc-Rac1 co-transfection efficiency). Rac1 could be activated by full-length E1A, but not by mut1, suggesting that E1A activates Rac1 through its N-terminal domain.

Despite multiple efforts, we did not detect a direct interaction between E1A and Rac1 by co-precipitation (data not shown). Therefore, we assumed that E1A might recruit intermediate components involved in Rac1 activation. Activity of small G-proteins such as Rac1 is regulated by guanine nucleotide exchange factors (GEFs), GTPase activating proteins (GAPs), and GDP dissociation inhibitors (GDIs) [30]. E1A might either enhance the activity of a GEF or inhibit a GAP or a GDI, thus enhancing loading with GTP or preventing GTP hydrolysis. GEF activity as well as inactivation of GDIs require the participation of ERM proteins [19-25]. The ERM protein family comprises three members with similar molecular structure and functions: Ezrin, Radixin, Moesin. siRNA-mediated knockdown of ERM proteins, either each of the three separately or all three combined, reduced E1A-dependent c-Jun activity (Fig. 4). Because we achieved only partial knockdowns, the suppression was also only partial. Although we have yet not been able to persistently detect a direct interaction between E1A and ERM proteins (data not shown), the conclusion is justified that ERM proteins (especially Ezrin and Radixin) are involved in c-Jun activation by E1A. Taking these results together, we suggest that E1A activates the JNK/c-Jun signaling pathway upstream of Rac1, and ERM proteins are involved in this activation.

## DISCUSSION

Our data demonstrate the existence of a new E1A pathway, which results in JNK-dependent activation of the transcription factor c-Jun. The pathway involves Rac1 and its downstream effectors MEKK1 and MKK4/7. One of the most important aspects of our current findings is that E1A exerts a rapid (in minutes) JNK signaling pathway activation. This novel action differs from the E1A-dependent mechanisms reported so far.

E1A is a nuclear protein and a known regulator of gene expression. Although E1A does not bind to DNA directly, it interacts with a large number of cellular proteins that are involved in the regulation of transcription [9, 10]. Activation of c-Jun may be the result of transcriptional regulation, e.g. the coactivator p300 can participate in transcription of the *jun* gene [31, 32]. E1A interaction with p300 is however clearly not involved in the activation of JNK as described here: the deletion mutants of E1A differentiate p300 from the new pathway. For instance, deletion of E1A amino acids 4-16 is critical for E1A-p300 interaction [26, 33], but did not cause a drop of c-Jun activation. Amino acids 17-25 are crucial for E1A-dependent activation of c-Jun (Fig. 2E), defining a sequence section of yet unknown interactor. The Rac1-to-JNK pathway can also be activated through a transcriptional event: E1A induces *de novo* expression of Tiam1, a Rac1 GEF, and reportedly leads to activation of Rac1 [34]. However, our immediate activation of the cascade did not involve new protein synthesis. Thus, possibly there are two ways to activate JNK and c-Jun by E1A: the fast activation described here, and a slow transcription- and Tiam1-dependent pathway. However, the latter, the delayed transcription-dependent increase of JNK activity, was not detected in our experiments.

Another, intriguing, mechanism of Rac1 activation has been reported: RACK1, an amazingly multifunctional scaffold protein recruits PLC, Src, and PP2A [35]. Through Src-dependent phosphorylation of the Rac1 GEF, Vav2, [36] or through the PI3K/Akt signaling pathway [37] RACK1 may address Rac1. It also interacts with MKK7 and enhances MKK7/JNK activity [38] and can even directly interact with JNK and promote its activation through phosphorylation by PKC [39]. Even more intriguingly, E1A reportedly interacts with RACK1 through the E1A N-terminus [40]. Although these data offer several possible mechanisms for a role of RACK1 in E1A-induced JNK/c-Jun activation, we have yet not seen a suppression of the cascade by downregulation of RACK1 (data not shown).

We demonstrated here that another promising family of scaffold proteins is involved in E1A-induced c-Jun activation. The ERM proteins, linked to both the plasma membrane and the actin cytoskeleton, are required in order to activate small G-proteins: direct association of ERM proteins with GEFs regulates their activity, leads to their localization at the plasma membrane, and scaffolds the assembling of GEFs with the small G-proteins [19-24]. ERMs also interact with GDIs and displace GDIs from the G-protein enhancing GTP loading [25, 41]. Although it is not yet clear how E1A makes use of the ERM proteins, we showed that their action is required for c-Jun activation by E1A.

Could E1A exert a non-nuclear function at all? ERM and RACK1 are cytoplasmic proteins, and Rac1 and its activating complex are associated with the plasma membrane. E1A contains a Nuclear Export Signal that is conserved in the C subgroup of adenoviruses [42] suggesting a possible translocation of E1A from nucleus to cytoplasm. A minor cytoplasmic occurrence of E1A and interactions with a number of cytoplasmic proteins have indeed been reported [43-46]. Thus, a cytoplasmic role of E1A in JNK/c-Jun activation would be plausible.

What could be the consequence of JNK activation by E1A? In rodent or human cells, E1A exerts a number of very different phenotypes, from transformation to apoptosis. There is an amazing similarity to the phenotypes linked to JNK/c-Jun activation. Like E1A, c-Jun stimulates proliferation and cooperates with oncogenic Ras in cellular transformation, participating in the induction of both anchorage-independent and autocrine growth [47, 48]. For tumorigenesis, c-Jun needs to be phosphorylated [49]. Not surprisingly, JNK is elevated in many tumors [50, 51], and is required for Ras-initiated tumor formation [52] and tumorigenesis *in vivo* [53]. On the other hand, again resembling E1A, the JNK/c-Jun signaling pathway is a major determinant of apoptosis and survival [54, 55] and JNK isoforms can act as tumor suppressors [56-58]. Such opposite outcome is likely due to the formation of different c-Jun dimers that target the expression of either proliferation-promoting or pro-apoptotic genes: by participating in several possible dimer combinations, c-Jun directs distinct transcriptional programs [55, 59, 60]. It is tempting to speculate that the diversity of cell fates induced by E1A in fact represents the diversity induced by c-Jun and JNK, and that at least part of the E1A-induced cellular phenotypes are mediated through JNK and c-Jun. Along these lines, E1A favors the expression of c-Jun:ATF2 target genes, e.g. *jun* itself [47] and cyclin A [61], and induces the expression of p14/p19^ARF^ [62], possibly through c-Jun.

The activation of JNK through the N-terminus of E1A could be instrumental for transformation. Interestingly, the E1A deletion mutant mut1, which cannot activate JNK, is defective in transforming cells in cooperation with oncogenic Ras [26, 63, 64]. To our knowledge, no attempts have yet been made to transform *jun^−/−^* or *jnk ^−/−^* cells by E1A. Our prediction is that these cells would be transformation-defective.

## MATERIALS AND METHODS

### Plasmids

To construct wild type and mutant E1A-ER fusion proteins, the ligand-binding domain of the human estrogen receptor alpha (amino acids 282-595) was excised from plasmid HE14 [65] and introduced between RSV-LTR and SV40 polyA of RSV-0 [11] to obtain RSV-ER. E1A-12S wild type or mutant sequences (deletion mutant d1101: deletion of amino acids at positions 4-25; d1102: positions 26-35; d1103: positions 30-49; d1104: positions 48-60; d1107: positions 111-123; d11108: positions 124-127; kindly provided by Stenley T. Bayley, McMaster University, Canada; [66]) were amplified by PCR using the primers 5'-CGGGTCGACGGACTGAAAATGAGACAT-3' and 5'TCGCCGGTCGACCAGCTAGCTGGCCTGGGGCGTTTACAGCTC-3' and cloned into the Sal-I site of RSV-ER upstream and in frame of the ER sequence. E1A deletions of amino acids at positions 4-14, 10-20, 15-25, 14-16, 20-22, 23-25, 26-28, 29-31, and 71-80 were generated from E1A-ER plasmid using the PfuUltra Polymerase kit (Stratagene, #600396). The tagged constructs HA-JNK1 and HA-MKK4, as well as the dominant-negative mutants of MKK4 and MEKK1 were kindly provided by Michael Karin (University of California, San Diego, USA); the myc-MKK7 and dominant-negative mutants of MKK7 were kindly provided by Lloyd Greene (Columbia University, USA); the tagged construct myc-Rac1 were kindly provided by Alan Hall (University College London, UK). Gal4-c-Jun and pG5.EfΔlux3 constructs were described earlier [67].

### siRNA

The down-regulation of RhoA, Rac1, Cdc42, and ERM proteins was carried out using small interfering RNA (siRNA), which were transfected using Lipofectamine 2000 (Invitrogen) according to manufacturer's instructions. SMARTpools (cocktails of four siRNAs) were purchased from Dharmacon (Thermo Scientific): against RhoA (#L-042634-00), Rac1 (#L-041170-00), Cdc42 (#L-043087-01), Ezrin (#L-046568-01), Radixin (#L-044428-01), Moesin (#L-047230-01), and control NonTarget (#D-001206-13).

### Antibodies

The antibodies against the following proteins were used: human ERα (Santa Cruz Biotechnology, #sc-543); HA (hybridoma clone 12CA5; kindly provided by Peter Angel, DKFZ, Germany); Rb (BD Biosciences, #554136); c-Jun (Cell Signaling Technology, #9165), phospho-c-Jun (Ser63) (Cell Signaling Technology, #9261), myc Tag (Upstate, #06-549); E1A (Calbiochem, #DP11-100UG; Santa Cruz Biotechnology, #sc-58658); RhoA (Cytoskeleton, #ARH03), Rac1 (Upstate, #05-389); Cdc42 (Thermo Scientific, #89857D), Ezrin (Cell Signaling Technology, #3145), Radixin (Cell Signaling Technology, #2636), Moesin (Cell Signaling Technology, #3150).

### Cell culture and transfections

NIH 3T3 cells (ATCC) were grown at 37°C and 5% CO_2_ on tissue culture dishes in Dulbecco's Modified Eagle's Medium (DMEM; Gibco BRL) containing 10% fetal calf serum (FCS) (Gibco BRL) supplemented with penicillin/streptomycin. Expression vectors encoding inducible E1A-ER fusion proteins were introduced into NIH 3T3 cells; stably transfected clones were obtained upon cultivation of the cells in the presence of neomycin (800 μg/ml). Both stable and transient transfections were performed using Lipofectamine 2000 (Invitrogen) according to manufacturer's instructions. E1A-ER-expressing cells were cultivated in phenol-red-free DMEM supplemented with steroid-free charcoal-stripped FCS. For the E1A activation, the cells were treated with 1 μM 17-β-estradiol (E_2_, Sigma-Aldrich) for the indicated periods of time.

### Immunoblotting

To prepare whole-cell extracts, cells were washed with the Tris-buffered saline (TBS) and lysed with a RIPA buffer containing 50 mM Tris-HCl (pH 7.4), 150 mM NaCl, 2 mM EDTA (pH 8.0), 1% Nonidet P-40, 0.1% SDS, 50 mM NaF, 1 mM Na_3_VO_4_, and a protease inhibitor cocktail (Boehringer Mannheim). Protein concentration was measured by Bradford assay. Proteins were resolved by SDS-PAGE and electrotransferred to a nitrocellulose membrane. The membrane was blocked in a 5% nonfat dry milk in TBS with 0.1% Tween 20 (blocking buffer) for 1 h and then incubated overnight at 4°C with primary antibodies and for 1 h at room temperature with the corresponding horseradish peroxidase (HRP)-conjugated secondary antibodies (DAKO) both diluted in the blocking buffer. Immunoreactive bands were visualized using ECL.

### Co-immunoprecipitation and metabolic labeling

For the detection of E1A-ER-associated proteins, cells were transfected with E1A-ER using Lipofectamine 2000 (Invitrogen). For methionine labeling, serum-starved cells were washed twice with warm phosphate buffered saline (PBS) and cultivated in medium without methionine, cystine, glutamine (Sigma-Aldrich) as well as without serum, but supplemented with 143 μCi/ml of ^35^S-pro-Mix (Amersham) for 4 h. Estradiol-treated transfected cells were washed with PBS and lysed in immunoprecipitation buffer containing 150 mM NaCl, 50 mM Tris-HCl (pH 8.0), 5 mM EDTA (pH 8.0), 1% Nonidet P-40, and a protease inhibitor cocktail (Boehringer Mannheim). 500 μg of protein from total lysate was incubated overnight with anti-human ERα antibodies, and then with protein A/G-agarose beads (Santa Cruz Biotechnology) for 2 h. Co-precipitated E1A-ER-associated Rb was detected by immunoblotting using specific antibodies. Immunoprecipitated ^35^S methionine labeled proteins were resolved by gradient SDS-PAGE and visualized by autoradiography.

### In vitro protein kinase assays

To measure the activation of JNK1, MKK4 and MKK7 by E1A-ER, NIH 3T3 cells were grown in 10 cm dishes and transiently co-transfected with plasmids encoding E1A-ER (20 μg) and HA-tagged JNK1 or HA-MKK4 or myc-MKK7 (10 μg each). To avoid activation of growth factor receptors, the cells were serum-starved for 24 h, then treated with 1 μM E_2_ for the indicated periods of time, washed with ice cold PBS, harvested and lysed with a buffer containing 20 mM Tris (pH 7.5), 137 mM NaCl, 2 mM EDTA, 1% Triton X100, 10% glycerol, 25 mM β-glycerophosphate, 1 mM Na_3_VO_4_, 1 mM PMSF, 25 mM NaF, 1 mM DTT, and a protease inhibitor cocktail (Boehringer Mannheim). 300-500 μg of protein from centrifuged supernatant was used for immunoprecipitation with appropriate antibodies together with protein A/G agarose beads (Santa Cruz Biotechnology). After 2 h of incubation at 4°C, the beads were washed twice with lysis buffer and once with kinase buffer (25 mM HEPES (pH 7.4), 25 mM MgCl_2_, 25 mM β-glycerophosphate, 1 mM DTT, 0.1 mM Na_3_VO_4_). The *in vitro* protein kinase assays were performed in 25 μl of kinase buffer containing 5 μCi of [γ-^32^P]ATP and 2 μg of either GST-c-Jun 1/166 or GST-JNK1. After 20 min of incubation at 30°C, Laemmli sample buffer was added and probes were boiled for 5 min. Then phosphorylated substrates were resolved by SDS-PAGE and visualized by autoradiography.

The non-radioactive *in vitro* protein kinase assay of total JNK on Fig. 1C was performed using KinaseSTAR JNK activity assay kit (BioVision, #K431-40). Briefly, cells were treated with 1 μM E_2_ for the indicated periods of time and lysed with a provided buffer. 250 μg of protein from total lysate was incubated with provided JNK antibodies for 45 min and then with protein A-Sepharose beads for 1 h at room temperature. Co-precipitated JNK was washed by provided buffer and incubated with c-Jun Protein/ATP Mixture for 4 h at 30°C. Then Laemmli sample buffer was added and probes were boiled for 5 min. 20 μl of the supernatant were resolved by 12% SDS-PAGE and then detected by immunoblotting using anti-phospho-c-Jun (Ser63) antibodies.

### c-Jun-GAL4 reporter assay

NIH 3T3 cells were transiently co-transfected using Lipofectamine 2000 (Invitrogen) with E1A-ER-coding, Gal4-c-Jun, pG5.EfΔlux3, Renilla luciferase (pRL-SV40, Promega) plasmids and either dominant negative mutants of JNK upstream signaling components or siRNA oligonucleotides. After the transfection, cells were cultivated in 10% of charcoal-stripped FCS for 24 h and then incubated with 1 μM E_2_ for the indicated periods of time. Specific JNK kinase inhibitor SP600125 (10 μM; Calbiochem) was added 5 min prior to E_2_ treatment. Firefly luciferase activity was determined and normalized to Renilla activity. Statistical analysis was performed using GraphPad Prism software.

### Pull-down of GTP-loaded Rac1

GTP-loaded Rac1 was pulled-down from lysates by GST-human Pak1-PBD in accordance with the manufacturer's instructions (Active Rac1 pull-down and detection kit, Pierce, #89856). Briefly, cells were transiently co-transfected with E1A-ER-coding and myc-Rac1 plasmids using Lipofectamine 2000 (Invitrogen), and in 24 h were treated with 1 μM E_2_ for 10 min and lysed with a provided buffer. 1 mg of protein from total lysate was incubated with Glutathione resin and GST-human Pak1-PBD for 1 h at 4°C. Co-precipitated GTP-loaded Rac1 was resolved by 12% SDS-PAGE and then detected by immunoblotting using anti-myc Tag antibodies.
